# Dinâmica da regionalização e repercussões dos vazios assistenciais na comercialização da saúde em municípios rurais remotos

**DOI:** 10.1590/0102-311XPT194523

**Published:** 2024-09-09

**Authors:** Adriano Maia dos Santos, Lígia Giovanella, Márcia Cristina Rodrigues Fausto, Lucas Manoel da Silva Cabral, Patty Fidelis de Almeida

**Affiliations:** 1 Instituto Multidisciplinar em Saúde, Universidade Federal da Bahia, Vitória da Conquista, Brasil.; 2 Escola Nacional de Saúde Pública Sergio Arouca, Fundação Oswaldo Cruz, Rio de Janeiro, Brasil.; 3 Universidade do Estado do Rio de Janeiro, Rio de Janeiro, Brasil.; 4 Instituto de Saúde Coletiva, Universidade Federal Fluminense, Niterói, Brasil.

**Keywords:** Regionalização da Saúde, Gestão em Saúde, Acesso aos Serviços de Saúde, Assistência Integral à Saúde, Serviços de Saúde Rural, Regional Health Planning, Health Management, Access to Health Services, Comprehensive Health Care, Rural Health Services, Regionalización, Gestión en Salud, Acceso a los Servicios de Salud, Atención Integral de Salud, Servicios de Salud Rural

## Abstract

Analisam-se a dinâmica da regionalização em municípios rurais remotos e as possíveis implicações dos vazios assistenciais na comercialização da saúde. Trata-se de um estudo de casos múltiplos, com abordagem qualitativa, por meio de 76 entrevistas semiestruturadas com gestores municipais, regionais e estaduais. Os resultados revelam que, particularmente nos estados da Região Norte, o desenho regional não repercutia a dinâmica social das populações e criava fluxos inadequados e rotas indesejadas. A agenda política municipal priorizava, muitas vezes, interesses díspares à regionalização e as questões da ruralidade não mobilizavam os gestores para a construção de um planejamento regional específico. Emendas parlamentares ocupavam um lugar imprescindível para o investimento em saúde e os gestores apontaram relações clientelistas para obter tais recursos, condicionada e corriqueiramente, pelo alinhamento político-ideológico. A escassez de serviços públicos favorecia a dependência do setor privado e a comercialização da saúde em diferentes situações. As grandes distâncias e a ausência de serviços públicos nas proximidades dos municípios rurais remotos tornavam a oferta do Sistema Único de Saúde (SUS) local eminentemente dependente do contrato com prestadores privados que negociavam no varejo ou por meio de pacotes de serviços. Por fim, na esteira das necessidades não atendidas e dos vazios assistenciais, nos municípios rurais remotos, agentes do mercado da saúde - empresas de fornecimento de insumos, consultorias, profissionais de saúde e serviços de transporte - ocupavam as brechas da provisão pública, algumas vezes controlando preços, oferta e disponibilidade dos serviços.

## Abstract (en)

This article analyzed the dynamics of regionalization in municipalities within hinterlands and the possible implications of gaps in care for the marketing of health. This is a multiple case study with a qualitative approach, involving 76 semi-structured interviews with municipal, regional, and state managers. The results show that, particularly in the Northern states, the regional scheme did not reflect the social dynamics of the populations and created inadequate flows and unwanted routes. The municipal political agenda often prioritized interests other than that of regionalization, and rural problems did not mobilize managers to build specific regional planning. Parliamentary amendments were essential for investment in healthcare and the managers pointed to clientelistic relationships to obtain such resources, often conditioned by political-ideological alignment. The scarcity of public services favored dependence on the private sector and the commercialization of health in different situations. The great distances and the lack of public services in municipalities in the hinterland made the local public health system offer eminently dependent on contracts with private providers who negotiated on a retail basis or via service packages. Lastly, in the wake of unmet needs and gaps in care in remote rural municipalities, players in the healthcare market ₋ companies supplying inputs, consultants, healthcare professionals, and transportation services ₋ filled the gaps in public provision, sometimes controlling prices, supply and availability of services.

## Abstract (es)

Se analizan las dinámicas de la regionalización en municipios rurales remotos y las posibles implicaciones de las brechas de la atención en la comercialización de la salud. Se trata de un estudio de caso múltiple, con enfoque cualitativo, que realizó 76 entrevistas semiestructuradas a los gestores del municipio, de la región y del estado. Los resultados mostraron que, principalmente en los estados de la Región Norte, el diseño regional no reflejó la dinámica social de las poblaciones y creó flujos inadecuados y rutas indeseables. La agenda política municipal frecuentemente priorizó intereses dispares a la regionalización y los temas de ruralidad no movilizaron a los gestores para construir una planificación regional específica. Las enmiendas parlamentarias fueron esenciales para invertir en sanidad, y los gestores señalaron las relaciones clientelares para obtener estos recursos, muchas veces condicionadas por el alineamiento político-ideológico. La escasez de servicios públicos favoreció la dependencia del sector privado y la comercialización de la salud en diferentes situaciones. Las grandes distancias y la falta de servicios públicos en las cercanías de municipios rurales remotos hicieron que la oferta del Sistema Único de Salud local dependiera eminentemente del contrato con proveedores privados que negociaban al por menor o mediante paquetes de servicios. Finalmente, ante las necesidades no cumplidas y las brechas de atención, en los municipios rurales remotos, los agentes del mercado de la salud (empresas proveedoras de insumos, consultorías, profesionales de la salud y servicios de transporte) ocuparon las brechas en la prestación pública mediante muchas veces el control de los precios, de la oferta y de la disponibilidad de los servicios.

## Introdução

O acesso a serviços de saúde para pessoas que vivem em territórios rurais, mesmo em países de renda alta ^1^ ^,^ ^2^, é comumente pior que em áreas urbanas ^3^. Populações que vivem em países com baixo e médio padrão de desenvolvimento acumulam problemas sócio-históricos que, em sinergia com o local de residência, intensificam essas iniquidades ^4^ decorrentes de lacunas na provisão e na qualidade da assistência na atenção primária à saúde (APS) ^5^, como insuficiência de serviços ambulatoriais e hospitalares ^6^ ou por dificuldades de acesso oportuno ^7^.

No Brasil, a ruralidade e as grandes extensões territoriais impactam no padrão sanitário e refletem as heterogeneidades socioespaciais engendradas no modo de ocupação do território, na distribuição dos bens e direitos do Estado ^8^. No caso da saúde, instituiu-se, no âmbito do Sistema Único de Saúde (SUS), em decorrência da pressão de movimentos sociais organizados, a Política Nacional de Saúde Integral das Populações do Campo, da Floresta e das Águas (PNSIPCFA) ^9^, que reconhece o modo de vida e a estreita relação com o meio ambiente de determinados grupos sociais historicamente invisibilizados, inclusive pelo SUS ^10^.

Não obstante exista um arcabouço político-institucional na perspectiva da universalidade, integralidade e equidade no SUS, as disparidades socioeconômicas e de saúde vigentes no país se agravam em cenários nos quais predominam a assimetria, a verticalidade, a competitividade e a fragilidade das relações multilaterais entre municípios, associados à limitada governança das autoridades sanitárias dos entes subnacionais em áreas remotas ^11^. Por sua vez, a organização de serviços em redes de atenção à saúde, via agrupamentos municipais, em territórios regionalizados com instâncias de decisão colegiada tem sido a modelagem nacional eleita para equacionar as assimetrias, mitigar a fragmentação e fomentar a cooperação interfederativa ^12^ ^,^ ^13^. Ainda assim, a autonomização municipal e as disputas políticas têm constrangido as relações solidárias, mesmo em situações de interdependência sanitária ^14^ e a estratégia de regionalização em curso no SUS permanece insuficiente para suplantar a miríade de desafios em regiões extensas ^15^ ^,^ ^16^, com populações vivendo em territórios rurais e remotos ^17^ ^,^ ^18^.

Entre os inúmeros problemas, a oferta e o acesso aos serviços na atenção especializada ^17^ ^,^ ^19^, a regulação assistencial ^20^ e o transporte sanitário ^7^ emergem como os principais obstáculos, especialmente entre municípios de pequeno porte, para conformação de uma região de saúde com capacidade para o cuidado integral ^21^. Por outra via, os consórcios públicos de saúde ascendem como estratégia para organização e provisão pública de serviços com destaque à atenção especializada ^22^, aquisição racional de insumos e medicamentos ^23^, impactando, inclusive, na oferta e preços cobrados por prestadores privados ^24^.

No contexto dos vazios assistenciais, da incipiente capacidade de oferta e regulação assistencial, as relações público-privadas modelam-se nos interstícios do SUS, uma vez que o Estado - com alta demanda - é o principal comprador dos serviços de saúde em cenários de extrema escassez na oferta ^19^. Tal discrepância estimula a comercialização da saúde diante do grande poder de negociação/barganha dos prestadores privados e empresários de insumos de saúde ^25^. Esse enredo, aliado ao subfinanciamento do SUS, impacta na capacidade dos municípios de prover adequadamente os serviços de saúde à população e, paradoxalmente, abre espaço para mecanismos que retroalimentam a comercialização dos serviços públicos ^19^ ^,^ ^26^, inclusive por meio de agentes do Estado, via emendas parlamentares ^27^, relações clientelísticas ^28^ e outras combinações que desvirtuam o interesse público e acentuam as desigualdades.

Este artigo analisa a dinâmica da regionalização em territórios conformados por municípios rurais remotos e discute possíveis implicações dos vazios assistenciais na comercialização da saúde.

## Aspectos metodológicos

**Figura 1:**
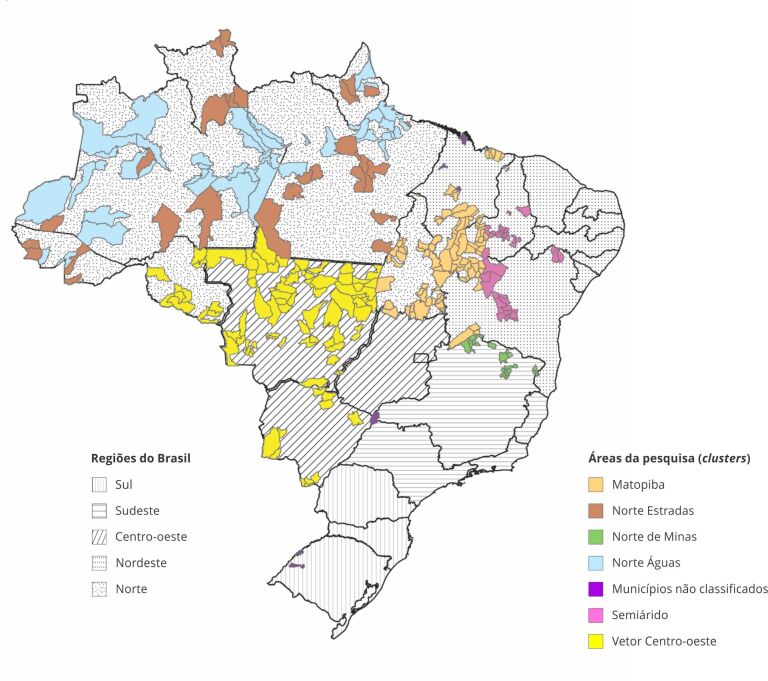
Mapa das áreas da pesquisa (*clusters*).

Este artigo analisa parte dos resultados da pesquisa *Atenção Primária à Saúde em Municípios Rurais Remotos no Brasil* ^29^. O estudo partiu da caracterização dos 323 municípios rurais remotos, segundo classificação do Instituto Brasileiro de Geografia e Estatística (IBGE) ^30^, que foram tipificados segundo características socioeconômicas, demográficas e de saúde e definidos seis *clusters* com lógicas espaciais específicas (Figura 1): Matopiba, Norte de Minas, Norte Águas, Norte Estradas, Semiárido e Vetor Centro-oeste ^29^.

A amostra de municípios foi intencional, estruturada a partir dos seis *clusters*, nos quais foram escolhidos dois ou mais municípios que corresponderiam ao “município tipo”, ou seja, com características socioeconômicas, demográficas e de saúde mais frequentes no conjunto dos municípios rurais remotos do respectivo *cluster*. Ao “município tipo”, agregou-se um ou mais municípios *outliers*. Ao final, foram selecionados 27 municípios rurais remotos distribuídos em dez estados - Acre, Amapá, Amazonas, Bahia, Maranhão, Mato Grosso, Minas Gerais, Pará, Piauí e Tocantins - e 17 regiões de saúde (Tabela 1) para realização de estudo de casos múltiplos, com abordagem qualitativa, por meio de entrevistas semiestruturadas ^29^.

**Tabela 1:** Indicadores socioeconômicos. Municípios rurais remotos, Brasil, 2019.

**Áreas da pesquisa (*cluster*)/Município (UF)**	População *	Região de saúde	PIB *per capita* (R$) *	Densidade demográfica (habitantes/km^2^) **	Índice de Gini **	População beneficiária do Bolsa Família (%) ***
Vetor Centro-oeste						
Nova Lacerda (Mato Grosso)	6.640	Pontes e Lacerda	6.699,08	1,32	0,51	31,15
Vila Bela da Santíssima Trindade (Mato Grosso)	16.128	Pontes e Lacerda	4.792,22	1,16	0,59	25,37
Tabaporã (Mato Grosso)	9.489	Juara	10.544,18	1,10	0,44	12,82
Matopiba						
Avelino Lopes (Piauí)	11.289	Chapada das Mangabeiras	1.696,78	8,81	0,56	89,04
Júlio Borges (Piauí)	5.627	Chapada das Mangabeiras	1.857,29	4,27	0,53	71,37
Monte Alegre do Piauí (Piauí)	10.613	Chapada das Mangabeiras	4.463,45	4,32	0,57	59,70
Redenção do Gurguéia (Piauí)	8.778	Chapada das Mangabeiras	2.033,69	3,50	0,57	90,49
Campos Lindos (Tocantins)	10.116	Médio Norte Araguaia	7.462,56	3,02	0,67	34,28
Formosa da Serra Negra (Maranhão)	19.089	Balsas	1.787,62	5,15	0,63	58,32
Tasso Fragoso (Maranhão)	8.521	Balsas	33.246,50	1,93	0,58	56,04
Norte de Minas						
Bonito de Minas (Minas Gerais)	11.230	Januária	1.498,67	2,78	0,57	54,57
Indaiabira (Minas Gerais)	7.351	Taiobeiras	1.907,11	7,49	0,48	56,98
Rubelita (Minas Gerais)	5.995	Salinas	2.179,29	6,11	0,50	73,26
Norte Águas						
Boa Vista do Ramos (Amazonas)	19.207	Baixo Amazonas (Amazonas)	1.796,14	7,14	0,59	52,16
Maués (Amazonas)	63.905	Baixo Amazonas (Amazonas)	1.928,86	1,56	0,64	71,10
Curuá (Pará)	14.393	Baixo Amazonas (Pará)	2.117,85	9,78	0,60	62,18
Prainha (Pará)	29.866	Baixo Amazonas (Pará)	2.332,63	1,97	0,68	75,23
Melgaço (Pará)	27.654	Marajó II	1.569,18	3,97	0,55	81,12
Vitória do Jari (Amapá)	15.931	Sudoeste	2.930,69	5,97	0,58	56,00
Aveiro (Pará)	16.388	Tapajós	1.741,61	0,93	0,60	48,80
Norte Estradas						
Jacareacanga (Pará)	41.487	Tapajós	12.523,54	0,15	0,69	28,88
Rurópolis (Pará)	50.510	Tapajós	1.708,94	6,99	0,57	27,82
Assis Brasil (Acre)	7.417	Alto Acre	3.026,10	1,40	0,61	75,98
Semiárido						
Ipupiara (Bahia)	9.865	Ibotirama	1.965,21	9,62	0,50	62,93
Morpará (Bahia)	8.519	Ibotirama	1.798,36	4,27	0,55	80,52
Pilão Arcado (Bahia)	35.048	Juazeiro	1.640,85	3,07	0,60	65,46
Rio Grande do Piauí (Piauí)	6.432	Vale dos Rios Piauí e Itaueiras	1.893,06	9,95	0,54	80,16

PIB: produto interno bruto; UF: Unidade da Federação.

* Estimativa populacional, competência 2019, segundo o Instituto Brasileiro de Geografia e Estatística ^48^;

** Segundo o Instituto Brasileiro de Geografia e Estatística ^49^;

*** Segundo o Ministério do Desenvolvimento Social ^50^.

Para compreender os desafios da dinâmica da regionalização em territórios rurais remotos e dos vazios assistenciais na comercialização da saúde, objeto deste artigo, foram analisadas 76 entrevistas com gestores da saúde. Os gestores estaduais foram selecionados em cada estado e corresponderam ao coordenador estadual da atenção básica ou cargo correlato (11 gestores). Os gestores municipais foram selecionados para cada município rural remoto participante e corresponderam aos secretários municipais de saúde e coordenadores locais da APS (53 gestores), havendo apenas uma recusa. Na regional de saúde, da qual fazia parte o município rural remoto visitado, priorizou-se entrevistar como gestor regional um técnico envolvido com o tema da APS em âmbito regional (12 gestores). Quando não existia a instância regional de saúde, buscou-se entrevistar um representante da Comissão Intergestores Regional (CIR), preferencialmente o coordenador.

As entrevistas foram presenciais, realizadas nos respectivos locais de trabalho dos participantes, no período de maio a outubro de 2019, com duração média de 2h30min, gravadas em áudio e transcritas na íntegra.

**Figura 2:**
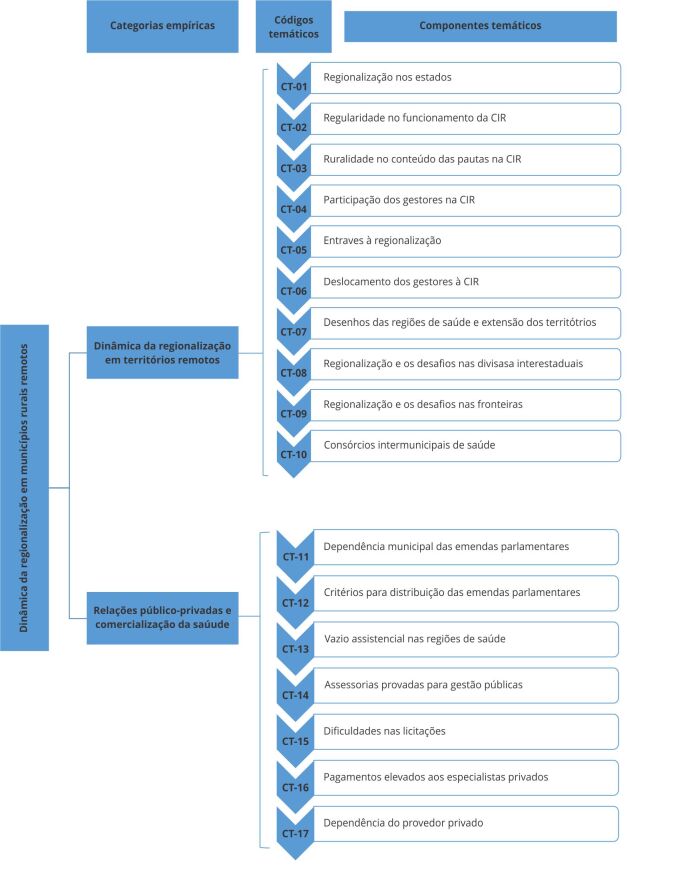
Fluxograma da análise temática sobre a dinâmica da regionalização em municípios rurais remotos do Brasil.

A interpretação dos dados foi orientada pela análise temática de conteúdo, identificando-se convergências e divergências. Realizou-se a ordenação dos dados a partir da leitura geral do material transcrito. O *corpus* foi ordenado e classificado, etapas em que as transcrições foram lidas exaustivamente e emergiram 17 componentes temáticos (CT) (Quadros 1 e 2). Na sequência, partiu-se para o cotejamento entre os CTs que, ao final, compuseram duas categorias temáticas: (i) dinâmica da regionalização em territórios remotos; e (ii) relações público-privadas e comercialização da saúde (Figura 2).

**Quadro 1:** Componentes temáticos e falas expressivas sobre a “dinâmica da regionalização” em territórios rurais remotos do Brasil.

COMPONENTES TEMÁTICOS	ESTRATOS DE FALAS DOS GESTORES ENTREVISTADOS
[CT-01] Regionalização nos estados	“*A discussão da regionalização de Minas é muito avançada. Como falei, a gente tem 77 regiões, 13 macrorregiões de saúde, e a regionalização da saúde é muito discutida aqui; a questão dos hospitais e dos serviços micro e macrorregionais, que devem funcionar a partir de uma vinculação à APS; e tem a CIB, CIR, CIRAS muito fortes. Então, essa discussão é muito forte aqui no estado*” (Gestor estadual 1, Minas Gerais). “*A gente quer que essa nova gestão* [estadual] *otimize esses espaços [regionais] que já vêm de muito tempo,* (...) *a organização é muito boa, tem CIR, tem Consórcio de Saúde, das 16, em 15 regiões, com quase 90% dos municípios consorciados. Com exceção da Baixada Cuiabana, as outras regiões de saúde têm consórcio. E tem escritório regional de saúde em todas as regiões de saúde, então tem CIR instalada nas 16 regiões de saúde e ativa*” (Gestor regional 2, Vetor Centro-oeste, Mato Grosso). “*Na região amazonas, na verdade, a regionalização só acontece no direito, pois de fato não acontece. Isso aí é em todas as regiões* (...)*. Pelas particularidades do estado, seria o estado nacional que mais precisaria dessa regionalização. Tem município que não vai* [via de acesso] *para município nenhum. Não dá para ser de uma regional que ele não conversa com outro município. É tão distante que não compensa para ele*” (Gestor municipal 1, Norte Águas, Amazonas). “*...está muito frágil, essa questão da regionalização.* (...) *para os próprios gestores, ainda não caiu a ficha de que vai ser regionalizado, mas ainda está muito tímida.* (...) *a atuação ainda é muito municipalizada. Até porque, o gestor maior* [prefeito] *pensa* (...) *que pode ser ofuscado pelo fulano e pelo beltrano* [gestor de outro município]*. Então, cada um quer fazer, mostrar mais, e aí a parte regional das discussões* [não avança]” (Gestor regional 1, Norte Estradas, Acre).
[CT-02] Regularidade no funcionamento da CIR	“*Todas CIR funcionam e têm uma agenda que é programada para o ano todo. A gente dá um apoio com os escritórios regionais, porque tem algumas questões que são resolvidas lá que vêm em forma de proposição operacional e aqui a gente tem que fazer o nosso parecer, para poder fazer a resolução para a CIB, fazer a minuta de resolução. Então, todas as CIR que tem, a gente se comunica com as áreas técnicas responsáveis pela APS no escritório regional para dar o apoio das proposições operacionais*” (Gestor estadual 1, Mato Grosso). “*...apesar de ter essa melhora toda em relação ao número de equipes e a cobertura, isso, às vezes, não se traduz nos indicadores, é isso que a gente vem trabalhando fortemente no COCAB* [reunião de coordenadores regionais da APS]*. Nesses dois anos, começamos a trabalhar o papel do coordenador* [da APS] *e suas atribuições. Porque, senão, ele acaba cooptado para gestão, naquela função de assessor de secretário*” (Gestor regional 1, Semiárido, Bahia).
[CT-03] Ruralidade no conteúdo das pautas na CIR	“*...não sei se por insegurança, não sei se por medo de perder o cargo, mas as pessoas ficam apáticas* [na CIR]*, ninguém briga com a sede* (...)*, se você contar do nosso colegiado, nós somos 20 municípios, se tirar quatro que falam alguma coisa, que tem coragem de brigar, de tentar repactuar, de tentar organizar, é muito*” (Gestor municipal 1, Norte Estradas, Pará). “*Às vezes, a gente discute as peculiaridades de cada um, mas como um desabafo*” (Gestor municipal 1, Vetor Centro-oeste, Mato Grosso). “[Debate específico sobre ruralidade?]*. Não vou mentir, não. Sabe por quê? Porque acaba o município assumindo isso*” (Gestor regional 2, Vetor Centro-oeste, Mato Grosso). “[A questão das populações rurais e as dificuldades de acessos] *é um tema que não percebo que isso é tratado. Parece um tema que acaba sendo normalizado, não é uma questão que mobiliza tanto nesse colegiado* [CIR]*. Eles falam muito sobre o SAMU* (...)*. Em relação a essas especificidades, eles vivenciam isso enquanto a município, mas não sinto que trazem para discutir no coletivo*” (Gestor regional 1, Semiárido, Bahia).
[CT-04] Participação dos gestores na CIR	“*...a sensação que a gente tem é que as coisas não andam por forças políticas partidárias.* (...) *são determinantes por não andar, a alta rotatividade de secretários* (...) *toda CIR, quando a gente vê, tem um secretário novo.* (...) *perdi as contas de quantas vezes já mudou de secretário,* [tem município que] *já tá no terceiro secretário.* (...) *então, como é que você dá continuidade,* (...) *aí, toda vez que aparece alguém novo, a gente tem que explicar tudo de novo*” (Gestor municipal 1, Norte Estradas, Pará). “*A CIR se reúne, mas muitos* [secretários de saúde] *não comparecem, porque estão cansados de nada ser resolvido.* (...) *tivemos a última reunião em Teresina, porque tivemos um congresso de secretários lá e aproveitamos para nos reunir*” (Gestor regional 1, Matopiba, Piauí). “*...faz tempo que não vou em CIR, sabe por quê? É só desgaste e nada funciona! Discussões que não têm continuidade* (...)*. Então, vou por uma questão, às vezes, que eu tenho que discutir e comungar com os outros municípios ou mando meu subsecretário que é meu representante na CIR, mas praticamente para fazer número, porque a estrutura do estado do Tocantins em relação à saúde está quebrada, falida*” (Gestor regional 2, Matopiba, Tocantins).
[CT-05] Entraves à regionalização	“*A gente tem uma história bem importante no processo de regionalização, mas ao longo do tempo e das mudanças de gestão, a gente foi perdendo um pouco essa capacidade que a gente tinha* (...) *os escritórios regionais, ao longo do tempo, percebemos que estão cada vez mais desestruturados, perdendo a sua capacidade de apoiar as regiões na tomada de decisão, enquanto que nós, também, enquanto SES, estamos perdendo essa capacidade de conduzir a política macroestadual e para dentro das regiões, também*” (Gestor estadual 1, Mato Grosso). “*Tivemos uma perda muito grande aqui, que foi a lei de estruturação administrativa.* (...) *éramos diretoria regional de saúde* (...)*, então, nosso trabalho, apesar das dificuldades, era muito mais fácil tendo um diretor em cada região de saúde do que como está agora. Agora, são três bases que fazem parte desse Núcleo Regional de Saúde e tem uma única coordenadora para dar conta de 37 municípios.* (...) *acabou interferindo no processo de regionalização*” (Gestor regional 1, Semiárido, Bahia). “*...temos regiões de saúde, não regiões administrativas.* (...) *não tem uma estrutura e não tem pessoas de referência direta do estado naquele local* [regiões de saúde]*, exceto nos hospitais,* [por exemplo] *quando vai fazer uma campanha de vacina, temos que conversar com 139 municípios* [individualmente]” (Gestor estadual 1, Tocantins).
[CT-06] Deslocamento dos gestores à CIR	“*...estamos a mais de 1.000km da capital, então, tudo pra gente é muito difícil. Então, chegar na CIB em Belém e o pessoal falar assim ‘ah, porque o pessoal do Oeste não aparece’. Como é que vou aparecer? Custa R$ 2.000,00, a passagem, não tem condição,* [esse valor] *dá pra comprar um monte de remédio, não vou gastar esse dinheiro indo pra Belém pra uma reunião. Então, isso que não entendem dentro do próprio estado, a dificuldade que temos de locomoção. Hoje, é mais barato ir pra São Paulo do que ir pra Belém, de passagem aérea*” (Gestor municipal 1, Norte Estradas, Pará). “*...para sair de Boca do Acre e ir para Manaus* [para uma reunião]*, ir e voltar, ele* [secretário de saúde] *gasta R$ 4.000,00, dentro do mesmo estado. É mais barato ir para São Paulo, a Brasília do que chegar na capital do estado, então tudo isso dificulta*” (Gestor estadual 2, Amazonas).
[CT-07] Desenhos das regiões de saúde e extensão dos territórios	“*...a Bahia tem uma extensão considerável, do tamanho da França...*” (Gestor estadual 1, Bahia). “*As distâncias entre os municípios são muito grandes, isso dificulta muito o acesso da população aos serviços de saúde, principalmente no quesito de problemas de média e alta complexidade. Para a gente conseguir estruturar esse fluxo aqui na região é algo bem desafiador. E dentro do próprio município também existe isso, temos municípios que que algumas localidades estão mais de 100km da sede, e isso de certa forma imprime uma necessidade de uma organização bem diferenciada*. (Gestor regional 1, Semiárido, Bahia). “*Claro que Almeirim e Faro são os municípios mais distantes da região do Tapajós e o acesso é mais difícil, geralmente dura 24 horas para chegar ao município. Jacareacanga, também, é uma terra muito distante, chega a ser 24 horas de viagem de carro*” (Gestor regional 1, Norte Águas, Pará). “*Porque o acesso realmente é difícil, temos muitas áreas ribeirinhas. Assim, áreas ribeirinhas no mesmo município que demora dois, três, quatro dias para chegar*” (Gestor estadual 1, Amapá). “*Quando chove, não tem condições de chegar nos ramais, os pacientes, às vezes, chegam carregados por redes. Temos que ter caminhonetes de tração para poder chegar e, muitas vezes, não conseguimos, pois, o veículo acaba atolando por ter muita lama. Eles ficam bem isolados. Há uns dois meses, fui buscar uma paciente e o carro só conseguiu chegar até certo ponto e a paciente teve que ser carregada durante duas horas para chegar até onde estávamos*” (Gestor municipal 2, Norte Estradas, Acre).
[CT-08] Regionalização e os desafios nas divisas interestaduais	“*‘X’ é o maior município do Maranhão, a gente tem ACS a 350km de distância daqui, numa região chamada Barra Funda.* (...) *na fronteira com a Bahia*” (Gestor regional 3, Matopiba, Maranhão). “*...a gente atende aqui o povo da Bahia, o povo de ‘X’* [distrito da Bahia] *vem pro hospital, porque é mais perto o acesso pra eles aqui, do que voltar pra lá. Tem um município que é bem na divisa, aí a maioria da população vem pra cá, é do município de ‘Y’, mas fica mais perto daqui* [município ‘Z’] *do que* [da sede] *de ‘Y’,* [então] *vem pra ser atendido aqui*” (Gestor municipal 1, Matopiba, Piauí). “*...temos municípios* [na região de saúde] *que ficam há quase 700km de distância* [sede da regional]*, então, isso também é uma característica que dificulta, porque em Rondolândia, o acesso deles é mais rápido para Rondônia do que em Mato Grosso*” (Gestor regional 1, Vetor Centro-oeste, Mato Grosso). “*...tem o município de Chaves, na Ilha do Marajó, que é do Pará, mas é mais perto do Macapá, do que do Pará*” (Gestor estadual 1, Amapá).
[CT-09] Regionalização e os desafios nas fronteiras	“*...temos 417km de fronteira com a Bolívia. É muito difícil. Acaba que a Bolívia consegue ser mais carente que a nossa região. E temos que atender elas que vêm de lá.* (...) *elas vão para comunidade que faz divisa. Lá, a gente tem um PSF,* (...) *e vão para ganhar seus filhos, fazer consulta, uso de medicamentos, vacinas. E como vamos dizer que não para uma população? E elas fazem esse processo itinerante, ficam dois meses para receber os benefícios do SUS e voltam para casa*” (Gestor municipal 1, Vetor Centro-oeste, Mato Grosso). “[Faz fronteira com] *Peru e uma parte da Bolívia. Então, nesse sentido, é muito difícil* (...) *nessa parte de saúde,* (...) *porque eles* [estrangeiros] *vêm buscar o atendimento no Brasil e a nossa população é específica. O SUS é universal, mas para aquela população* [do Brasil]*; o recurso que vem é calculado em cima de X população, não considera uma fronteira, então, já não é mais universal, ele é limitado aos munícipes daquele município.* (...) *muitos têm dupla nacionalidade, adquire o cartão SUS, mas continuam morando lá.* (...) *e a dificuldade que encontro nessa parte é essa: as doenças que não têm fronteira!*” (Gestor regional 1, Norte Estradas, Acre).
[CT-10] Consórcios públicos de saúde	“*...então, tudo o que a gente quer comprar, é mais barato pelo consórcio. Então, atende um monte de especialidade dentro do consórcio, compramos o serviço, mas atende lá...*” (Gestor municipal 2, Vetor Centro-oeste, Mato Grosso). “*...aqui tão tentando articular uma capacitação pros municípios pra fazer o consórcio de medicamentos pra comprar todo mundo por consórcio. Só que a pessoa que vinha dar esse treinamento e explicar direitinho como funcionaria, nunca veio e a gente já chegou a ir pra Belém, mas ela não pode nos receber*” (Gestor municipal 1, Norte Estradas, Pará. “*No final de 2017, a gente inaugurou quatro policlínicas, depois, no início de 2018, mais quatro, e esse ano, está previsto mais oito policlínicas, essas policlínicas são regionais.* (...) *as policlínicas são via consórcio de saúde constituído de municípios, onde o estado faz parte.* (...) *só teve quatro municípios que não queriam fazer parte da policlínica.* (...) *porque tinha desconfiança* [dos prefeitos]*. Alguns acharam que isso não ia acontecer, pagaram pra ver e, aí, o que acontece é que tá funcionando, tá acontecendo, a população tem feito avaliações muito positivas e, aí, eles estão voltando pra renegociar, se inserir nas policlínicas que são mais próximas deles*” (Gestor estadual 1, Bahia). “*Os consórcios são pactos entre gestores; o benefício para os municípios é que conseguem comprar em grande quantidade* [por] *um valor menor e têm questões que, às vezes, são muito burocráticas para o município atender com certa urgência que, pro consórcio, ele consegue nos atender.* (...) *é estipulado um valor a cada município e dentro do consórcio tem as taxas administrativas que são divididas entre os consorciados, mas ganhamos, porque consegue comprar e licitar valores menores*” (Gestor regional 1, Norte de Minas, Minas Gerais).

ACS: agentes comunitários de saúde: APS: atenção primária à saúde; CIB: Comissão Intergestores Bipartite; CIR: Comissão Intergestores Regional; CIRAS: Comissões Intergestoras Regionais Ampliadas; COCAB: Colegiado de Coordenadores de Atenção Básica; PSF: Programa Saúde da Família; SAMU: Serviço de Atendimento Móvel de Urgência; SES: Secretaria Estadual de Saúde; SUS: Sistema Único de Saúde.

**Quadro 2:** Componentes temáticos e falas expressivas sobre as “relações público-privadas e comercialização da saúde” em territórios rurais remotos do Brasil.

COMPONENTES TEMÁTICOS	ESTRATOS DE FALAS DOS GESTORES ENTREVISTADOS
[CT-11] Dependência municipal das emendas parlamentares	“*Teve algumas emendas parlamentares que o prefeito conseguiu. Aí, deu para equipar algumas* [unidades de saúde]*, deu para comprar alguns materiais, equipar com a estrutura física mesmo, ar-condicionado, essas coisas, até os móveis, mobília para os postos*” (Gestor municipal 1, Norte Águas, Pará). “*Hoje, 80% dos municípios* [do Brasil] *dependem diretamente da emenda parlamentar. Nosso estado depende 100%. Ninguém consegue resolver nada só com recurso próprio. Só consegue fazer algo se for com emenda*” (Gestor regional 2, Norte Águas, Amapá). “*...os municípios têm lançado muito mão dos recursos de emendas parlamentares,* (...) *isso auxilia o município na manutenção das equipes.* [Às vezes] *é o estado que também articula com os deputados, principalmente federais, para que direcionem a emenda parlamentar pra nós; e a gente faz a aquisição de equipamentos para concessão aos municípios.* (...) *então, os deputados negociam um milhão para o estado e, aí, já apontando quais os municípios que devem ser beneficiados e a gente faz a aquisição. A gente consegue comprar com preço bem mais barato, o município acaba recebendo a sessão dos equipamentos, carro, equipamentos de saúde bucal, entre outros pra estruturar as unidades*” (Gestor estadual 1, Bahia).
[CT-12] Critérios para distribuição das emendas parlamentares	“*...as emendas são feitas completamente sem critério, porque a gente dá um apoio mais não fica aqui no setor, mas um tempo atrás, fui ver uma emenda e era um município lá da região Noroeste - 40 armários de aço para atenção primária! O que a atenção básica vai fazer com 40 armários de aço? Quem é que fez isso aqui? Sabe, são umas coisas meio absurdas e, assim, o fluxo não exige uma aprovação técnica, o município faz o projeto e, muitas vezes, não é o município que faz o projeto, é o político que diz...*” (Gestor estadual 1, Mato Grosso). “*...nesse sentido, acho uma situação degradante a tal da emenda impositiva; agora, a gente só consegue* [recurso] *se for emenda, se o seu perfeito for bem relacionado, se ele for lá e puxar saco de A e de B, ele consegue, se não for, você não tem nada...*” (Gestor municipal 1, Norte Estradas, Pará). “*Normalmente tem um deputado que disponibiliza essas emendas para os municípios* (...)*. Normalmente já vem de lá destinado. Hoje, precisaríamos mais para custeio, aí, já vem de lá determinado para que fim será utilizado essa emenda*” (Gestor municipal 1, Norte de Minas, Minas Gerais). “*...os deputados encaminham recurso considerável, um recurso mais global, mas ele quer a prerrogativa de selecionar para seus municípios. O valor que a gente compra* [um equipamento] *é muito inferior ao que o município iria comprar. Então, acaba sendo racional a utilização do recurso e, também, a gente negocia com os parlamentares, o que a gente analisou que é estruturante; agora, onde ele vai investir, aí, é decisão dele.* (...) *tem agora a parte dos equipamentos eletrocardiograma, que é pra viabilizar o telediagnóstico. Então, a gente já vai pros parlamentares: ‘gostaria que vocês investissem nesse kit de equipamentos’. Eles olham, vou querer que vocês comprem pra esses municípios; a gente vai e compra e acaba estruturando.* (...) *se a gente deixar solto, quem vai estruturar a equipe de saúde é o parlamentar, da cabeça dele e dos técnicos dele*” (Gestor estadual 1, Bahia).
[CT-13] Vazio assistencial nas regiões de saúde	“*A meu ver, a barreira maior, e aí não estou no olhar só da APS, estou pensando na Rede, em conjunto, a barreira maior é justamente os encaminhamentos; a necessidade de um serviço especializado*” (Gestor estadual 1, Minas Gerais). “*A maioria dos casos deveria ser de neurologia, mas vai terminar na psiquiatria por causa da dificuldade com o neurologista. Porque não tem, mesmo em Imperatriz, mesmo São Luís é uma demanda* [elevada]*, até particular é difícil.* (...) *São Luís está a 1.000km. Difícil a nossa situação; aqui é o extremo sul e São Luís é o extremo norte do estado*” (Gestor municipal 2, Matopiba, Maranhão). “*O mais grave é a disponibilidade de especialistas, que ainda estão muito centralizados, e ainda temos pouco profissionais que atuam nessas regiões* [de saúde]*, a maioria dos especialistas estão nos grandes centros, então, inevitavelmente, as pessoas que moram nessas localidades* [municípios rurais remotos] *precisam se deslocar aos grandes centros pra poder ter atendimento especializado.* (...) *nosso problema hoje na Bahia, se chama exame de patologia, porque temos raros patologistas na Bahia,* [aliás] *isso é no Brasil inteiro*” (Gestor estadual 1, Bahia).
[CT-14] Assessorias privadas para gestão pública	“*...não tem só ela* [empresa de assessoria privada]*, mas é a maior que tem.* (...) *eles cresceram, porque deixamos que crescessem, pois, a gente não conseguiu dar o apoio ali nas regiões para os municípios e os municípios recorreram a empresas privadas. Houve falha nossa como secretaria, porque não conseguiu coordenar a política, porque não consegue dar o apoio de qualidade. Inclusive, um dos donos da empresa já foi gestor de um município muito importante da região sul mato-grossense, e depois veio ser gestor aqui da SES, e aí continuaram com a empresa, e obviamente que a empresa cresceu ainda mais depois disso*” (Gestor estadual 1, Mato Grosso). “*...tiramos dúvidas, assessoria jurídica, todos os setores eles têm uma pessoa responsável, tanto que, uma compra que você tá em dúvida se você pode fazer, eles te dão o parecer no atendimento. Agora mesmo, dia 14 e 15, eles vão vir dar duas oficinas que eu solicitei, eles vieram semana passada dar uma oficina para os conselheiros de saúde. Então, estão bem presentes, eu não trocaria, a gente renovou contrato deles, falei para o secretário que não troco, não vou mexer no que tá dando certo, porque a gente tem poucas pessoas trabalhando aqui na gestão, então não daríamos conta, essa é a realidade, se não tivéssemos alguém* [empresa] *pra nos dar esse suporte.* (...) *vai ter audiência pública da prestação de contas agora em agosto, daí eu mando todas as informações* (...)*, eles fazem essa montagem* [dos slides] *e traz para mim no dia da apresentação, e tenho também a minha opção: ou apresento ou eles apresentam*” (Gestor municipal 2, Vetor Centro-oeste, Mato Grosso).
[CT-15] Dificuldades nas licitações	“*...tem algumas licitações que* (...) *ninguém se interessou.* [Fornecedores] *falam que é pouco dinheiro e que não vêm. A gente fica refém* (...) *teve uma licitação de equipamentos pra maternidade, que apareceu uma única empresa e aí a empresa põe o preço que quer.* (...) *cada município faz a sua licitação, a empresa que aparece é que leva e as empresas grandes, não têm interesse*” (Gestor municipal 1, Norte Estradas, Pará).
[CT-16] Pagamentos elevados aos especialistas privados	“*Temos uma psiquiatra, a qual pago preço de ouro, ainda tenho que mandar buscar em Floriano* [350km]*. O suporte que ela me dá é fazendo o ambulatório no CAPS-AD.* (...) *outros secretários municipais ficam me pedindo para arrumar vagas no cardiologista - que vêm uma vez por mês -, por exemplo, para usuários deles, mas não tenho vagas nem pra atender os meus. Então, ou manda tudo para o privado ou, quando não manda, o pessoal não possui acesso*” (Gestor regional 1, Matopiba, Piauí). “*O nosso médico sai muito caro aqui, por 15 dias, eles ganham R$ 66.000,00 cada um. Ou paga, ou não tem. Gasto mensalmente no hospital R$ 500.000,00, R$ 300.000,00 é com médico.* (...) *a gente consegue, porque tem pactuação com a hidrelétrica, royalties, essas coisas*” (Gestor municipal 3, Norte Estradas, Pará).
[CT-17] Dependência do provedor privado	“*Os pequenos municípios* [a prestação] *é muito mais privada, pois os municípios maiores conseguem ter uma estrutura mais adequada, podem lançar mão de algum tipo de credenciamento, mas a gestão é pública, e nos casos dos municípios pequenos ainda tem essa questão de contrato de empresa, ou contratualização com empresas ou profissionais privados*” (Gestor estadual 1, Bahia).

APS: atenção primária à saúde; CAPS-AD: Centro de Atenção Psicossocial Álcool e Drogas; SES: Secretaria Estadual de Saúde.

CIR: Comissão Intergestores Regional.

O conjunto de dados contém as características essenciais do universo pretendido e seus resultados agregam elementos representativos e passíveis de transferibilidade ^31^ em outros cenários nacionais similares.

A pesquisa foi aprovada pelo Comitê de Ética em Pesquisa da Escola Nacional de Saúde Pública Sergio Arouca da Fundação Oswaldo Cruz (ENSP/FIOCRUZ; parecer nº 2.832.559), com anuência dos municípios.

## Resultados

### Dinâmica da regionalização em territórios remotos

A conformação de agrupamentos de municípios em regiões de saúde foi uma realidade constatada em todos os estados incluídos na pesquisa, independentemente do *cluster*. Ainda assim, a consolidação dos mecanismos de gestão regional e de compreensão dos gestores sobre a regionalização foi bastante heterogênea, com importantes avanços nos estados de Minas Gerais (*cluster* Norte de Minas), Mato Grosso (*cluster* Vetor Centro-oeste) e Bahia (*cluster* Semiárido), e acentuada fragilidade institucional nos estados do Norte (*clusters* Norte Águas e Norte Estradas) do país - Acre, Amapá, Amazonas e Pará [CT-01].

A CIR foi uma instância colegiada que funcionava com regularidade em todas as regiões de saúde, embora o conteúdo dos debates, segundo os gestores, fosse ritualístico e centrado prioritariamente na aprovação de projetos de incentivo federal para a implantação de serviços municipais. Problemáticas comumente debatidas foram a regulação assistencial, a oferta de vagas de serviços de alto custo e a organização dos serviços hospitalares nas respectivas regiões de saúde. Da mesma forma, ainda que a APS devesse estar articulada à rede intermunicipal, somente na Bahia foram constatados espaços de discussão em âmbito regional por meio do apoio institucional e compartilhamento de experiências, numa perspectiva de fortalecer a porta de entrada de forma solidária [CT-02].

As questões da ruralidade não mobilizavam os gestores para a construção de um planejamento regional específico e, quando surgiam nas pautas, tinham um caráter de desabafo e queixa em relação aos problemas identificados. Para alguns entrevistados, na CIR, muitos gestores mostravam-se apáticos, não faziam os enfrentamentos necessários, dada a assimetria de poder historicamente estabelecida entre os municípios, e ninguém contra-argumentava o gestor da sede da região de saúde. Ademais, praticamente todos os gestores afirmaram desconhecer a PNSIPCFA. Entre os poucos que sinalizaram conhecê-la, não havia direcionamento específico - municipal ou regional - das ações em saúde para tais grupos populacionais. As particularidades decorrentes da ruralidade eram desarticuladas e tratadas a depender da disposição de cada gestor [CT-03].

Além disso, a formação e a rotatividade de secretários de saúde comprometiam a continuidade das pautas na CIR. Havia, ainda, a participação seletiva de alguns gestores nas reuniões, pois só frequentavam a CIR quando havia assuntos de seu interesse. Um gestor municipal frisou a discrepância das pautas apresentadas pela Secretaria de Estado da Saúde (SES) à CIR, sobretudo por desconsiderar as especificidades municipais e centrar as cobranças em metas inatingíveis, independentemente do contexto locorregional [CT-04].

Mesmo em um estado que foi referência nacional no processo de regionalização como o Mato Grosso, o gestor estadual ressaltou que houve desmobilização e perda de protagonismo ao longo dos anos, entre outras coisas, por conta do enfraquecimento dos escritórios regionais, a redução do quadro técnico, além da própria regionalização, que nem sempre é uma prioridade dos gestores. De forma convergente, a função dos escritórios regionais nas regiões de saúde foi destacada como impasse para o apoio aos municípios rurais remotos em todos os estados (*clusters*), sobretudo pelo sucateamento dessas instâncias - baixo financiamento e quadro reduzido de trabalhadores. Alguns estados, apesar das dimensões, não tinham escritórios regionais e os profissionais da SES, localizada na capital, necessitavam deslocar-se aos diferentes territórios para algum apoio direto [CT-05].

Nos estados com extensões continentais, como Amazonas e Pará (*clusters* Norte Águas e Norte Estradas), a participação dos gestores municipais nas instâncias colegiadas - Comissão Intergestores Bipartite (CIB) e CIR - era comprometida pelos longos deslocamentos e restrição de recursos financeiros. No Pará, algumas reuniões da CIR eram realizadas na capital, fora da região de saúde, para aproveitar a reunião da CIB e, assim, viabilizar a participação de todos os gestores. Essas distâncias refletiam, também, na inadequação dos desenhos das regiões de saúde, especialmente no Amazonas, Pará (*clusters* Norte Águas e Norte Estradas) e Mato Grosso (*cluster* Vetor Centro-oeste), cujas distâncias ou o valor do transporte entre o município rural remoto e a sede da regional poderia ser maior que o deslocamento para o estado vizinho ou mesmo para a capital do estado, em decorrência do fluxo dos rios e da disponibilidade de transporte. Nesse sentido, os gestores sugeriram que o debate deveria ser, também, interestadual para compatibilizar outras peculiaridades geográficas [CT-06].

A contiguidade territorial não poderia ser o único parâmetro diante da dimensão territorial e das especificidades de deslocamento, porquanto cheias e vazantes dos rios criavam outras lógicas distintas às burocráticas de gabinete. Precisava-se, segundo os gestores, de um planejamento regional ascendente e que incorporasse os fluxos reais em cada território. No Tocantins (*cluster* Matopiba) e em algumas regiões da Bahia (*cluster* Semiárido), por exemplo, as reuniões da CIR eram itinerantes entre os municípios da mesma região de saúde. Por sua vez, o Amazonas e o Pará (*clusters* Norte Águas e Norte Estradas) englobavam, por exemplo, os municípios mais extensos do país - maiores, inclusive, que alguns países europeus. De modo geral, as dimensões ou as vias de deslocamentos - por terra ou água - dos estados/municípios investigados impunham importantes obstáculos na regionalização dos territórios [CT-07].

As diferentes extensões e formas de deslocamentos entre os estados do país agravavam as desigualdades e acentuavam as barreiras de acesso aos serviços de saúde dentro de um mesmo território. Alguns municípios localizados na divisa com outros estados recebiam demandas do estado vizinho, mas não havia planejamento e nem acordos interestaduais para organizar tais fluxos que acabavam sendo informais e sem pactuação [CT-08].

Ademais, um aspecto importante no planejamento regional dos estados do Norte (*clusters* Norte Águas e Norte Estradas) e do Centro-oeste (*cluster* Vetor Centro-oeste) foi a questão das fronteiras com países vizinhos. Gestores do Acre, Amapá, Amazonas, Pará e Mato Grosso destacaram a ausência de políticas transnacionais que prevejam e apoiem os municípios e estados em relação à vigilância epidemiológica de arboviroses, cobertura vacinal, uso abusivo de álcool e outras drogas, acompanhamento de gestantes, entre outros problemas sociais e sanitários [CT-09].

Por fim, diante das inúmeras dificuldades de acesso e oferta restrita de serviços especializados nas regiões de saúde, intensificados em municípios rurais remotos, o consórcio público de saúde foi considerado por alguns gestores como uma estratégia para contornar ou minimizar obstáculos relacionados à escala das compras de insumos e à contratação de serviços. No Mato Grosso, por exemplo, os consórcios, implantados a partir de 1995 e ampliados ao longo do tempo, apareceram no relato dos gestores como um importante arranjo organizativo para provisão de atenção especializada e hospitalar nas várias regiões de saúde, ainda que com heterogeneidade no cardápio de serviços e na capacidade de provisão. Constatou-se, também, a experiência recente de consórcios na Bahia para policlínicas com indução do ente estadual e forte adesão dos municípios. Ainda assim, observavam-se dissonâncias de gestores municipais que não aderiram imediatamente ao consórcio em função de desavenças político-partidárias. Tratava-se de um tema controverso, pois gestores municipais temiam perder o protagonismo/poder locorregional se não fossem os provedores diretos do serviço em seu próprio território. Em Minas Gerais, também ocorriam experiências consorciadas nas regiões de saúde, mas, predominantemente, sem a participação do ente estadual [CT-10].

O Quadro 1 contempla os CTs sobre a dinâmica da regionalização em territórios remotos.

### Relações público-privadas e comercialização da saúde

Emendas parlamentares como modalidade de alocação de recursos públicos em saúde apareceram, frequentemente, no discurso de todos os gestores. Emendas parlamentares ocupavam um lugar imprescindível para investimento em saúde para os municípios rurais remotos - construção/reforma de estabelecimentos de saúde, aquisição de equipamentos, insumos e veículos, inclusive para o custeio de ações e serviços [CT-11].

A grande ponderação dos gestores pautava-se na ausência de critérios técnicos na distribuição ou no uso inadequado dos recursos oriundos das emendas parlamentares. Muitas vezes, os gestores acabavam adquirindo insumos “desnecessários” ou “não-prioritários” e, não raramente, a escolha do que seria adquirido partia do próprio político. Os gestores apontaram relações clientelistas para obtenção de tais recursos, condicionada corriqueiramente pelo alinhamento político-ideológico. Em síntese, as emendas criaram uma forte dependência municipal e uma relação estreita com forças político-partidárias locorregionais que interferiam no planejamento da saúde. Em alguns casos, como na Bahia, o gestor estadual buscava articular com os deputados o direcionamento mais racional das emendas, sem interferir, contudo, na escolha do município contemplado [CT-12].

Paralelamente, os municípios rurais remotos convivem com situações de vazios assistenciais, especialmente para a atenção especializada no SUS, dificuldades para a atração de profissionais, sobretudo de médicos especialistas, e para a composição de equipe técnica com formação adequada para a gestão. Esse cenário de escassez favorecia a dependência do setor privado e a comercialização da saúde em diferentes situações, sobressaindo as dificuldades de acesso para a população [CT-13].

Municípios rurais remotos do Mato Grosso (*cluster* Vetor Centro-oeste), por exemplo, contratavam uma assessoria e consultoria privada, com sede na capital, para apoiar a gestão nas mais variadas ações - planejamento, processos de tomada de decisão, implantação, adequação e manutenção de sistemas de informação, gerenciamento de rede em todos os níveis de atenção, auditoria de processos, regulação de serviços e implantação de programas de saúde. A assessoria poderia ser customizada conforme necessidade ou capacidade de pagamento de cada município e elaborava os instrumentos de gestão - Plano de Saúde, Relatórios Anuais de Gestão -, além de prestação de contas, avaliação de desempenho, entre outros. Em algumas circunstâncias, ficava responsável pelas apresentações em sessões públicas. Muitas vezes, o responsável pela assessoria era um ex-secretário de saúde que, após acúmulo de experiência pública, oferecia o serviço aos municípios via iniciativa privada [CT-14].

As licitações também eram desafios permanentes e os municípios rurais remotos ficam em desvantagem por questões de escala de compras e do afastamento geográfico dos centros distribuidores. Assim, os grandes fornecedores não tinham interesse em participar das licitações e, consequentemente, as empresas que se habilitaram cobravam preços abusivos, acima do praticado no mercado e, por vezes, não entregavam os produtos licitados. Os municípios rurais remotos ficavam reféns do mercado de equipamentos/insumos de saúde e necessitavam desembolsar recursos financeiros que comprometiam o orçamento público. Por outro lado, os municípios, com raras exceções, ainda que na mesma situação, não se articulavam para compras em conjunto e o fracionamento das aquisições gerava gastos elevados, comprometendo a capacidade de aquisição e provimento [CT-15].

A ausência de especialistas inflacionou os preços do mercado médico e tornou os ganhos bastante elevados para os profissionais que desejassem atuar em municípios rurais remotos ou mesmo nas sedes das regiões de saúde mais afastadas, sendo uma situação especialmente crítica nos *clusters* Norte Águas e Norte Estradas. A pouca oferta e todos os desafios geravam pagamentos elevados - um anestesista, por exemplo, podia ganhar até R$ 60 mil a cada quinzena. Os médicos, por sua vez, atuavam como “empresa” na modalidade de pessoa jurídica, frequentemente por ser mais vantajoso, uma vez que poderiam atuar em diferentes municípios e acumular rendimentos [CT-16].

As grandes distâncias e a ausência de serviços públicos nas proximidades dos municípios rurais remotos tornavam a oferta do SUS local eminentemente dependente do contrato com prestadores privados que negociavam no varejo ou por meio de pacotes de serviços - consultas, exames e procedimentos - com as secretarias de saúde. Por outro lado, os gestores adquiriam tais serviços mesmo majorados, a depender do local cujo serviço público fosse ofertado via pactuação - sede da região de saúde ou capital do estado -, diante da distância, do tempo de deslocamento, da necessidade de acompanhante, entre outros. Em algumas circunstâncias, o recurso financeiro requerido para o translado do paciente tornava-se maior que o valor do serviço de saúde, o que incentivava a compra direta, pelo gestor municipal, em clínicas ou profissionais na região [CT-17].

O Quadro 2 exemplifica os CTs sobre as relações público-privadas e comercialização da saúde.

## Discussão

A heterogeneidade no processo de regionalização ^32^ expressa a capacidade técnica bastante frágil nos territórios mais remotos e a intencionalidade na agenda política municipal, muitas vezes reticente ao compartilhamento solidário na forma de organizar e gerir o sistema de saúde locorregional ^14^ ^,^ ^17^.

A racionalidade dos Planos Diretores Regionais definiu, em cada estado, as regiões de saúde e buscava agregar os diferentes municípios a um território sanitário, frequentemente por contiguidade entre os limites municipais, muito embora gerasse iniquidades por desconsiderar barreiras geográficas e econômicas dos territórios rurais e remotos. Ainda assim, a lógica da região de saúde intenciona conformar um espaço geográfico com capacidade para oferta integrada e diversificada de ações e serviços de saúde, atentando-se para a escala e o escopo em cada ponto e nível de atenção, otimizando os deslocamentos entre local de residência e o prestador do serviço, por meio de redes de atenção à saúde ^33^. Entre os inúmeros desafios, a indisponibilidade de meios de locomoção para acesso aos pontos de atenção, na região de saúde ou fora dela, implica em desfechos sanitários desfavoráveis e iniquidades, especialmente, aos residentes em áreas remotas ^7^. De tal modo, há necessidade de dispositivos compensatórios e reparatórios aos espaços e populações que sofrem efeitos adversos do desenho regional para mitigar possíveis iniquidades.

Nos territórios estudados, particularmente os estados da Região Norte, o desenho regional não repercutia a dinâmica social das populações e criava fluxos inadequados e rotas indesejadas e, por isso, era criticado por gestores regionais e municipais. Em comum, todos os municípios rurais remotos estão localizados em regiões de saúde com baixa oferta de serviços, com insuficiência da provisão pública de serviços ambulatoriais e hospitalares, embora coincida, também, aos locais com os piores indicadores de leitos e médicos por habitante, ou seja, reflete uma dupla ausência - desinteresse privado e insuficiência dos recursos públicos próprios para a oferta adequada ^32^. Em situação menos desfavorável, em decorrência da expansão do agronegócio e de *commodities*, alguns municípios rurais remotos dos *clusters* Matopiba e Vetor Centro-oeste atraem serviços privados, embora, no conjunto, reproduzam as adversidades sanitárias comuns aos demais casos ^29^.

As instâncias colegiadas funcionavam com reuniões regulares, embora a agenda política municipal priorizasse, muitas vezes, interesses díspares à regionalização. A dinâmica da CIR tornava-a um recinto burocrático - discussões pontuais e reativas - com pautas induzidas por políticas nacionais e estaduais com insuficiência de diálogo sobre as causas dos problemas da região. Nesse sentido, a complexidade dos contextos sociossanitários, a persistência e a recorrência de problemas históricos, aliadas à morosidade das políticas de saúde em âmbito regional impactavam na adesão dos secretários às reuniões colegiadas.

Em âmbito nacional, as CIR são um componente capilarizado de governança do SUS, estabelecidas como espaços de consolidação do pacto federativo setorial, embora pautem-se em debates emergenciais da gestão em detrimento de um planejamento regional de médio e longo prazo ^34^ ^,^ ^35^. Não por acaso, os gestores não conheciam ou incorporavam, por exemplo, a PNSIPCFA ^9^, apesar de possuírem territórios vastos com populações vivendo remotamente e em situação de ruralidade ^29^.

A formação profissional e a rotatividade dos gestores interferiam ora na compreensão, ora na continuidade dos temas debatidos em plenária, subaproveitando a CIR. Em decorrência disso, muitos deles omitiam sua opinião, cedendo espaço aos gestores com alguma formação na saúde, com longo tempo na função e acúmulo de saber da experiência ou àqueles com maior capital político, geralmente da sede regional. Em instâncias colegiadas do SUS, os conflitos decorrentes de assimetrias entre os entes federados perpassam todo processo de negociação e pactuação; assim, a disputa por aporte financeiro, diante do subfinanciamento, gera acirramento das tensões nas relações intergestores ^36^ e paradoxos entre necessidades regionais e interesses locais ^37^.

A sinergia entre desenho regional inadequado, territórios extensos e desinteresse em compor efetivamente a CIR impactava os municípios mais dependentes de uma política de saúde solidária e interdependente. Nesse sentido, os processos de decisão, planejamento e avaliação em estruturas gerenciais policêntricas impõem grandes desafios políticos - negociação e geração de consensos, regras de atuação, distribuição e partilha de recursos e mecanismos de decisões colegiadas ^38^. Tais atributos para a gestão de redes regionalizadas são complexos e requerem práticas democráticas no processo de tomada de decisão das políticas públicas que, paradoxalmente, podem ser mais incipientes em territórios cunhados pelo clientelismo arraigado nas relações de acesso aos bens e serviços coletivos, que combinam traços contraditórios como a desigualdade e a solidariedade ^28^.

As fronteiras entre países e as divisas estaduais somam-se às complexidades territoriais na negociação intergestores para a integração dos serviços de saúde, num contexto de assimetrias na organização dos sistemas e serviços. Embora representassem situações menos comuns no conjunto das experiências analisadas, o setor de saúde é estratégico para mitigar entraves ao desenvolvimento das diversidades sub-regionais requerendo um planejamento integrado nas fronteiras ^39^ e em espaços interestaduais ^40^. Nas faixas de fronteira, constatou-se a força atrativa do SUS e o consequente tencionamento dos sistemas municipais em função da população flutuante que, embora utilize os serviços públicos, não é contabilizada para os repasses financeiros.

As emendas parlamentares tornaram-se “moeda de troca” entre as instâncias governamentais - nacionais e subnacionais ^41^. Na experiência dos gestores, as emendas parlamentares desempenhavam, por vezes, o principal recurso para investimento, muito embora, as alianças entre executivo municipal e parlamentares estivessem permeáveis aos interesses e às práticas clientelistas, com frequente distorção na alocação do financiamento público. O destino da emenda forjado em cenário de pouca transparência e baixa regulação - na apresentação, na aprovação ou na execução - foi poucas vezes questionado pelos entrevistados, tornando-se um artifício de benefícios recíprocos entre gestores-parlamentares, mas com perdas relativas aos cidadãos. Nesse sentido, as emendas funcionam como mecanismo para deslocamento direto de recursos aos municípios de forma pulverizada e, frequentemente, fisiológica, num efeito conflitante ao planejamento integrado e locorregional ^27^. Além disso, as emendas parlamentares, dentro dos diferentes estados brasileiros, concentram-se em municípios que menos precisam de repasse, ou seja, a alocação orçamentária dos deputados não corresponde à carência municipal por recursos para a saúde ^42^.

Nas regiões de saúde, a cooperação interfederativa para a construção de arranjos organizativos que respondam às necessidades impostas pela integralidade à saúde entre entes subnacionais autônomos e assimétricos permanece como um desafio a ser equacionado em todo Brasil ^43^. Nessa seara, entre os estados investigados, havia importantes experiências de consórcios na Bahia ^44^, em Minas Gerais ^45^ e no Mato Grosso ^15^ com repercussão no fortalecimento recíproco dos municípios consorciados com apoio estadual. Ademais, os consórcios forjam um importante arranjo institucional no SUS para o fortalecimento da regionalização ^46^, especialmente com a coordenação, o aporte financeiro do ente estadual e o simultâneo resguardo da autonomia municipal ^22^ ^,^ ^44^, inclusive contribuindo, por meio da provisão pública, para refrear a segmentação e a mercantilização da saúde ^26^. Não obstante, os consórcios estão distribuídos heterogeneamente no país, contraditoriamente distantes das necessidades dos municípios rurais remotos e de outros territórios com severa dificuldade na oferta de serviços especializados, concentrando-se nos estados do Sul e Sudeste ^47^.

Na esteira das necessidades não atendidas e dos vazios assistenciais, nos territórios remotos, agentes do mercado da saúde - empresas de fornecimento de insumos, consultorias, profissionais de saúde e serviços de transporte - ocupavam as brechas da provisão pública, algumas vezes articulando-se para atuar em “cartéis”, controlando preços, oferta e disponibilidade dos serviços. Nesses casos, havia a submissão do ente público à mercantilização da saúde que, paradoxalmente, era retroalimentada por recursos públicos.

Tais ambiguidades sinergicamente nutriam, direta ou indiretamente, uma teia de comercialização da saúde. Assim, as grandes distâncias fomentavam uma rede de transportes privados de passageiros, mais cara para as localidades mais isoladas e carentes. Os serviços especializados, por sua vez, seguiam a mesma lógica invertida e, assim, por serem mais escassos, eram negociados frequentemente por médicos avulsos que podiam cobrar valores exorbitantes. Por fim, tais tramas funcionavam como um círculo vicioso, uma vez que toda essa mercantilização da assistência à saúde comprometia os recursos dos municípios rurais remotos, gerando menor capacidade de investimento municipal justamente nos territórios mais empobrecidos. Os elevados valores pagos aos serviços de saúde pelos gestores públicos permitiam o empresariamento médico e, consequentemente, tornavam desinteressante a vinculação ou credenciamento dos profissionais com o SUS.

Tal fenômeno resulta das relações imbricadas entre agentes públicos e privados que aproveitam as oportunidades geradas, pela escassez na oferta e pelas extensões geográficas, e articulam-se via opções de linhas de financiamento público para, intencionalmente, assegurarem a segmentação e a comercialização da saúde ^26^.

## Considerações finais

Estudos de abrangência nacional tendo o foco em municípios rurais remotos e sua dinâmica locorregional para oferta de serviços públicos são exíguos, particularmente tendo a regionalização como espectro de análise. As regiões de saúde formam importantes arranjos organizativos para consecução de uma rede pública interdependente; porém, os vazios assistenciais e as desigualdades subjacentes aos municípios rurais remotos imprimem paradoxos entre necessidades públicas regionais e interesses locais/individuais, sobretudo quando o desenho institucional desconsidera a dinâmica e os fluxos dos distintos territórios - vias de trafegabilidade, contiguidades territoriais, populações tradicionais etc.

Da mesma maneira, a dinâmica das relações público-privadas e as repercussões dos vazios assistenciais na comercialização da saúde em municípios rurais remotos pouco divergiram entre os *clusters*, com algum destaque de maior presença de serviços privados nos territórios mais “iluminados” em decorrência da expansão do agronegócio em municípios dos *clusters* Matopiba e Vetor Centro-oeste, embora reproduzam as adversidades sanitárias comuns aos demais casos.

Nesse enredo, o subfinanciamento do SUS aumenta a dependência por alocações diretas de recursos de emendas parlamentares e, consequentemente, cristaliza o personalismo e as alianças políticas na gestão pública, muitas vezes em detrimento da equidade e da justiça social, produzindo desigualdades evitáveis. Por sua vez, diante da escassez na provisão pública direta, o repasse via emendas parlamentares estimula contratos públicos com prestadores privados que, num segundo momento, retroalimentam a comercialização da saúde e a teia de interesses recíprocos na interface público-privada.

Nas regiões analisadas, a cobertura de planos privados e o desembolso direto são menos incidentes e abrangentes, entre outros motivos, por conta do baixo poder aquisitivo da população e do desinteresse de prestação direta privada em baixa escala para populações dispersas em áreas extensas. Portanto, o SUS é o principal prestador direto e comprador de ações, serviços e insumos da iniciativa privada, requerendo, para mitigar os interesses conflitivos, a regulação pública via comissões/colegiados intergestores e amplo controle social. Essa, por conseguinte, foi uma importante lacuna constatada nos *clusters* investigados em todos os estados.

O caráter monopsônico do SUS em estados e regiões de saúde poderia mitigar a investida privada por meio da oferta pública direta e aquisições compartilhadas de insumos/serviços entre os municípios, reduzindo a atomização das compras no varejo, de modo a ampliar a escala e o escopo dos serviços para viabilizar a integralidade assistencial, com melhor custo-benefício para produzir justiça sanitária com equidade. No estudo, os consórcios públicos de saúde, particularmente a existência de policlínicas com oferta direta e financiamento contínuo e compartilhado entre gestores municipais e estaduais, mostraram-se um arranjo organizativo com potência para reequacionar as assimetrias locorregionais na oferta da saúde e atenuar as desigualdades em municípios rurais remotos no Brasil.

Em última instância, a ausência de uma política nacional para a atenção especializada no SUS, o desfinanciamento e a descontinuidade de importantes políticas de saúde nos últimos anos - acentuados entre 2016 e 2022 - impõem a retomada imediata e consistente de investimentos com maior expressão em territórios com populações historicamente vulnerabilizadas.
